# Safety and efficacy of Cerebrolysin in early post-stroke recovery: a meta-analysis of nine randomized clinical trials

**DOI:** 10.1007/s10072-017-3214-0

**Published:** 2017-12-16

**Authors:** Natan M. Bornstein, Alla Guekht, Johannes Vester, Wolf-Dieter Heiss, Eugene Gusev, Volker Hömberg, Volker W. Rahlfs, Ovidiu Bajenaru, Bogdan O. Popescu, Dafin Muresanu

**Affiliations:** 1 Shaare Zedek Medical Center, Jerusalem, and Sackler Faculty of Medicine, Tel Aviv University, Tel Aviv, Israel; 2 Russian National Research Medical University and Moscow Research and Clinical Center for Neuropsychiatry, Ul. Donskaya 43, Moscow, 115419 Russia; 3 Department of Biometry and Clinical Research, IDV Data Analysis and Study Planning, Konrad-Zuse-Bogen 17, 82152 Krailling, Germany; 4 Max Planck Institute for Metabolism Research, Gleueler Street 50, 50931 Cologne, Germany; 5 Russian National Research Medical University, Moscow, Russia; 6 Department of Neurology, SRH Gesundheitszentrum Bad Wimpfen GmbH, Bad Wimpfen, Germany; 7 Department of Neurology, “Carol Davila” University of Medicine and Pharmacy, Bulevardul Eroii Sanitari 8, 050474 Bucharest, Romania; 8 Laboratory of Molecular Biology, “Victor Babes” National Institute of Pathology, Bucharest, Romania; 9 Department of Clinical Neurosciences, “Iuliu Hatieganu” University of Medicine and Pharmacy, Victor Babes Street No. 8, 400012 Cluj-Napoca, Romania; 10 ”RoNeuro” Institute for Neurological Research and Diagnostic, 37 Mircea Eliade Street, 400364 Cluj-Napoca, Romania

**Keywords:** Cerebrolysin, Stroke, Recovery, Early benefit, NIHSS, Meta-analysis

## Abstract

This meta-analysis combines the results of nine ischemic stroke trials, assessing efficacy of Cerebrolysin on global neurological improvement during early post-stroke period. Cerebrolysin is a parenterally administered neuropeptide preparation approved for treatment of stroke. All included studies had a prospective, randomized, double-blind, placebo-controlled design. The patients were treated with 30–50 ml Cerebrolysin once daily for 10–21 days, with treatment initiation within 72 h after onset of ischemic stroke. For five studies, original analysis data were available for meta-analysis (individual patient data analysis); for four studies, aggregate data were used. The combination by meta-analytic procedures was pre-planned and the methods of synthesis were pre-defined under blinded conditions. Search deadline for the present meta-analysis was December 31, 2016. The nonparametric Mann-Whitney (MW) effect size for National Institutes of Health Stroke Scale (NIHSS) on day 30 (or 21), combining the results of nine randomized, controlled trials by means of the robust Wei-Lachin pooling procedure (maximin-efficient robust test), indicated superiority of Cerebrolysin as compared with placebo (MW 0.60, *P* < 0.0001, *N* = 1879). The combined number needed to treat for clinically relevant changes in early NIHSS was 7.7 (95% CI 5.2 to 15.0). The additional full-scale ordinal analysis of modified Rankin Scale at day 90 in moderate to severe patients resulted in MW 0.61 with statistical significance in favor of Cerebrolysin (95% CI 0.52 to 0.69, *P* = 0.0118, *N* = 314). Safety aspects were comparable to placebo. Our meta-analysis confirms previous evidence that Cerebrolysin has a beneficial effect on early global neurological deficits in patients with acute ischemic stroke.

**Electronic supplementary material:**

The online version of this article (10.1007/s10072-017-3214-0) contains supplementary material, which is available to authorized users.

## Introduction

Early neurological improvements after acute ischemic stroke may have an impact of successful long-term functional recovery. While immediate thrombolysis has become a gold standard in very early acute ischemic stroke treatment, subsequent neuroprotective treatments failed to provide clear evidence so far [1]. However, the mechanism of action of pharmacological multimodal agents like Cerebrolysin is not limited to neuroprotection only, as its main capacity is to modulate post-lesional endogenous brain recovery. A recent meta-analysis, comparing Cerebrolysin to placebo in two studies of identical design (CARS-1 and CARS-2) [2], showed promising results on the National Institutes of Health Stroke Scale (NIHSS) at day 21 (MW 0.59, *P* = 0.0010; NNT = 7.1). The primary objective of the present meta-analysis was to assess whether these findings can be confirmed by a broader ensemble of randomized, placebo-controlled, clinical trials using identical methodology (population, intervention, comparator, outcome—PICO: “does 30 to 50 ml Cerebrolysin treatment, initiated within 72 hours post acute ischemic stroke and administered for at least 1 week, have an effect on early neurological status”). The deadline for information sources was December 31, 2016.

## Methods

### Protocol and registration

This meta-analysis follows the Preferred Reporting Items for Systematic Reviews and Meta-Analyses [3]. The nonparametric approach and the method of synthesis were operationalized under blinded conditions in the final statistical analysis plan of study CARS-2 (2014). A separate review protocol has not been prepared for this meta-analysis and the meta-analysis has not been included in any study registry since the objective of this meta-analysis was to verify the findings of the previously published meta-analysis on early neurological benefit (CARS-1, CARS-2) [2], using identical methodology.

### Eligibility criteria

Randomized, double-blind, placebo-controlled, completed clinical studies assessing efficacy of Cerebrolysin as add-on treatment to standard care of ischemic stroke were considered as eligible for inclusion in this meta-analysis if 30 to 50 ml Cerebrolysin were administered for at least 1 week and treatment was initiated within 72 h post-stroke. No restrictions were placed on language, publication (year, type, or status), study endpoint (duration, length of follow-up, type of outcome measures), or treatment intervention (treatment window, dosage, frequency, duration). Eligible studies published as abstract only were not included in this meta-analysis. Studies that did not provide outcome data or data usable for the meta-analysis as well as studies that did not meet the inclusion criteria were excluded. Primary outcome measure for the meta-analysis was the NIHSS. Efficacy was assessed at day 30 (or 21) with Last Observation Carried Forward (LOCF) replacement of missing values.

### Information sources

Information was sourced from the online databases MEDLINE (1946 to December 2016), CENTRAL (1948 to December 2016) and Cochrane Database of Systematic Reviews (1995 to December 2016), the German library collection of DIMDI (German Institute of Medical Documentation and Information, https://www.dimdi.de/static/en/index.html), which covers inter alia EMBASE (Excerpta Medica Database, 1947 to December 2016), SciSearch (1974 to December 2016), Biosis Previews (1926 to December 2016), ISTP (Index to Scientific and Technical Proceedings) and ISSHP (Index to Social Sciences & Humanities Proceedings, 1978 to December 2016), DARE (Database of Abstracts of Reviews of Effects, 2002 to March 2015) and PsycINFO (1966 to December 2016), the Swiss library collection ETH Bibliothek (http://www.library.ethz.ch/en/) covering inter alia Academic OneFile (1980 to July 2015) and Health Reference Center Academic (1980 to December 2016) and the US based WorldCat (https://www.worldcat.org/). In order to identify further completed studies, all references mentioned in the Cochrane review^35^ have been screened as well as study registries (ClinicalTrials.gov, https://clinicaltrials.gov/; Stroke Trials Registry, http://www.strokecenter.org/trials/; ISRCTN registry, http://www.isrctn.com/). Authors of abstracts or unpublished but registered studies have been contacted for further information. In addition, EVER Neuro Pharma has helped us to identify further sources. Searches were performed in December 2016 with the last search done on December 31, 2016.

### Search

The search terms “Cerebrolysin” and “stroke” were applied to all electronic database searches. The search strategy for MEDLINE was (“cerebrolysin”[Supplementary Concept] OR “cerebrolysin”[All Fields]) AND (“stroke”[MeSH Terms] OR “stroke”[All Fields]). No filters were used.

### Study selection

The title and the details of the periodical (if available) of the retrieved records have been listed on an Excel spreadsheet and screened by two independent researchers in order to remove identical records. The title and the abstract (if available) of the remaining records were scrutinized and obviously irrelevant reports have been excluded. We arranged for the complete reports of the remaining references and for professional translation services if published in languages other than English. After examination of the full text reports, potentially relevant studies have been identified and all related records were promoted to the stage of data extraction. Studies identified in registries of completed or unknown status were scrutinized for eligibility and cross-checked with retrieved citation. The flow of information is presented in the Online Supplement, Fig. X1.

### Data collection process

This meta-analysis combines the results of the nine double-blind, placebo-controlled studies by formal meta-analysis procedures based on individual patient data (IPD) and published aggregate data:

#### Studies with IPD

- Qaragozli 2011 [4]
- CASTA (Heiss et al. 2012) [5]
- CERE-LYSE-I (Lang et al. 2013) [6]
- CARS-1 (Muresanu et al. 2016) [2, 7]
- CARS-2 (Guekht 2015) [2, 8]

#### Studies with published aggregate data and/or study reports

- MRI-1 [9]
- MRI-2 [10]
- Amiri-Nikpour et al. 2014 [11]
- Xue et al. 2016 (individual patient data provided by authors for dropout inclusion) [12]

First-line analysis was the combination of all studies by means of a mixed meta-analysis approach [13] integrating results from IPD re-analyses as well as from aggregate data from publications and/or study reports. For one multicenter, multinational trial (MRI-1) [9], only Russian sites were published in a Russian journal. For the present meta-analysis, *all* sites were included capturing the aggregate data from the integrated clinical study report. For study Xue 2016 [12], raw data of dropouts were provided by the authors in order to allow LOCF analysis. Besides the sources listed above, no further data have been provided by authors of abstracts or unpublished but registered studies. An overview of data extraction is also provided in the Online Supplement, Table X2.

### Statistical analysis

#### Statistical methodology of nonparametric meta-analysis

The pre-planned nonparametric procedure was the well-known and robust Wilcoxon-Mann-Whitney test (WMW) [14–17]. The effect size measure associated to the WMW test is the Mann-Whitney (MW) measure of superiority, a highly robust effect size measure with minimized assumptions, representing the gold standard for full-scale ordinal analysis [18–22].

The technical expression for the MW is [*P*(*X* < *Y*) + 0.5 *P*(*X* = *Y*)]. The traditional benchmarks for the MW effect size measure are [23, 24]: 0.29 = large inferiority, 0.36 = medium inferiority, 0.44 = small inferiority, 0.50 = equality, 0.56 = small superiority, 0.64 = medium superiority, 0.71 = large superiority.

#### Handling of the original outcome scales

The original outcome scale available in all nine selected studies was the NIHSS at day 30 (or 21) [25]. The NIHSS scores were evaluated as changes from baseline at day 30 (or 21) by means of the robust MW effect size measure [19]. Further details on data handling are provided in the Online Supplement, Table X3.

The analysis of the secondary endpoint, the modified Rankin Scale (mRS), was performed for evaluation of the long-term outcome (day 90). The analysis was based on final changes from baseline (pre-planned nonparametric analysis) as well as on final Rankin scores with adjustment for NIHSS baseline severity (parametric sensitivity analysis).

Deaths, and patients with at least one treatment-emergent adverse event (TEAE) and at least one treatment-emergent serious adverse event (TESAE), were analyzed based on odds ratios (OR). In case of zero events in one of the treatment groups, the Peto odds ratio was calculated [26]. In two studies, no information was available on TESAE [4, 11]; in one study no information was available on TEAE [11]. These studies were omitted from the corresponding analysis.

For comparison with published results, the safety measures were additionally analyzed based on risk ratios (RR).

The rate of missing NIHSS values as compared to randomized subjects was below 10% in eight out of nine trials, thus well fulfilling the criteria for class I studies. For the five IPD studies [2, 4–8] and for one study with aggregate data [12], replacement of missing values was performed by means of the LOCF technique (for Xue 2016, the authors provided requested raw data in order to allow LOCF imputation). In three studies, only observed cases (OC) data were available for NIHSS evaluation [9–11]; however, the missing rates were well comparable between Cerebrolysin and placebo (7/67 vs. 8/66).

The primary neurological scale (NIHSS) was evaluated at day 30 (or 21) to verify the results of a previous meta-analysis on early neurological improvements [2]. This point in time also allowed to include the largest possible number of RCTs with availability of NIHSS (9/9). Final global disability was evaluated as secondary endpoint by means of the mRS at day 90.

For all studies, the ITT population was defined as the primary analysis population in the original investigational plans (with either LOCF or OC treatment of missing values). Thus, the original ITT population was also used as the basic patient population for the formal meta-analyses.

#### Method of synthesis

The pre-planned method of synthesis for the primary MW effect size measure [18–22] and for the RD was the Wei-Lachin test of stochastic ordering (one-dimensional test) [27], a maximin-efficient robust test (MERT) [28, 29], which provides a combined MW estimate and test of overall treatment effect from an ensemble of independent studies.

Further details on methods of synthesis are provided in the Online Supplement, Table X4.

## Results

### Study selection

The initial set of identified records through database search (*n* = 993) and other sources (*n* = 56) was adjusted for duplicates resulting in a total of 481 records. Of these, 421 records were excluded as they referred to reviews (*n* = 134) or meta-analyses (*n* = 4), experimental studies (*n* = 115), studies not related to Cerebrolysin (*n* = 20) or ischemic stroke (*n* = 67), studies with a non-interventional design (*n* = 3), case studies (*n* = 1), or open-label studies (*n* = 63). Eleven records were excluded that covered one study not controlled by placebo, nine studies that were published as abstract only, and one entry into an encyclopedia. In addition, three records obtained from study registries were excluded as one of these studies was never started according to the information obtained from the investigator and two completed studies did not provide results. The remaining 60 records covered 30 full-text articles of which 14 articles were excluded since they did not provide usable data (*n* = 9) or referred to studies that did not meet the inclusion criteria (*n* = 5) in terms of treatment start or dosage. The remaining 16 full-text articles referred to a total of 9 individual clinical trials which were included in this meta-analysis (for detailed flow chart of study selection see Online Supplement, Fig. X1).

### Study characteristics

Characteristics of the included studies are provided in Table 1. All studies had a prospective, randomized, double-blind, placebo-controlled design. They provided analysis data of 1879 patients of both genders and within an age range of 18–88 years; the number of patients in the individual studies ranged from 40 to 1070 patients. All nine studies provided day 21/30 data for NIHSS. Study quality was assessed independently by two members of the IDV methodology group (IDV Data Analysis and Study Planning, Krailling, Germany), who assigned a Jadad score of ≥ 3 for all trials. Disagreements were resolved by consensus. Main inclusion criteria were confirmed hemispheric ischemic stroke in the MCA territory or arterial branches of the internal carotid artery, and in most of these studies, stroke was of moderate to moderately severe intensity and Cerebrolysin treatment was started within 12 h post-stroke. Risk of bias assessment (see Online Supplement, Table X5) was performed using all available data (publications, clinical study reports, individual patient data files, feedbacks from authors). Attrition assessment was based on the primary point in time of this meta-analysis (21 or 30 days), not on later follow-up visits. For some studies, there was insufficient information available to permit judgment of all risks of bias. As several meta-analytic approaches have been performed in order to assess the robustness of the primary outcome parameter (NIHSS) of this meta-analysis, none of these studies was excluded.

**Table 1:** Overview of included studies evaluating clinical efficacy of Cerebrolysin in acute ischemic stroke

Source	Total no. of random. patients^c^	Cerebrolysin intervention	Comparator	Treatment initiation	Primary endpoint	Countries	NIHSS baseline
MRI-1	*N* = 60	10 or 50 ml/day for 10 days	Placebo (0.9% saline)	Within 12 h	MRI infarct volume at day 30	Russia, Romania	13.1^a,d^ 12.6^a^
		+ 100 mg ASA/day for 10 days					
		+ 250 mg ASA/day for 90 days + pentoxifylline (days 1–21: 300 mg, days 22–90: 800 mg/day)					
MRI-2 (Shamalov et al. 2010)	*N* = 47	50 ml/day for 10 days	Placebo (0.9% saline)	Within 12 h	MRI infarct volume at day 30	Russia	7.7^a^ 8.6^a^
		+ 100 mg ASA/day for 10 days					
Qaragozli 2011	*N* = 100	Day 1–7: 30 ml/day Week 2–4: 10 ml/day, 5 days/week	Placebo (0.9% saline)	Within 18 h	NIHSS at day 30	Iran	9.1^a^ 11.1^a^
		+ basic therapy					
CASTA (Heiss et al. 2012)	*N* = 1070	Cerebrolysin 30 ml/day for 10 days	Placebo (0.9% saline)	Within 12 h	Composite of NIHSS, mRS, BI at day 90	China, Hong Kong, South Korea, Myanmar	9^b^ 9^b^
		+ 100 mg ASA/day for 90 days					
CERE-LYSE-I (Lang et al. 2012)	*N* = 119	Cerebrolysin 30 ml/day for 10 days	Placebo (0.9% saline)	Immediately after rt-PA infusion	mRS at day 90	Austria, Croatia, Czech Republic, Slovakia, Slovenia	12.3^a^ 11.0^a^
		+ rt-PA over 60 min		Within 3 h			
Amiri-Nikpour et al. 2014	*N* = 46	Cerebrolysin 30 ml/day for 10 days	Placebo	Within 6–24 h	NIHSS at day 30, 60, 90	Iran	14^b^ 14^b^
		+ 100 mg ASA					
CARS-1 (Muresanu et al. 2016)	*N* = 208	Cerebrolysin 30 ml/day for 21 days	Placebo	Within 24–72 h	ARAT at day 90	Romania, Ukraine, Poland	9.1^a^ 9.2^a^
		+ basic therapy					
CARS-2 (Guekht 2015)	*N* = 240	Cerebrolysin 30 ml/day for 21 days	Placebo	Within 24–72 h	ARAT at day 90	Russia	7.5^a^ 6.8^a^
Xue et al. 2016	*N* = 84	Cerebrolysin 30 ml/day for 10 days	Placebo	Within 12 h	NIHSS and BI day 30	China	13.3^a^ 12.7^a^
			NBP				
		+ basic therapy					

^a^Means (Cerebrolysin vs. placebo)

^b^Medians (Cerebrolysin vs. placebo)

^c^All randomized groups

^d^50 ml group

### Results of individual studies

Effect sizes of individual studies are provided for all meta-analyses, with the respective confidence intervals, the *p* values, and the sample sizes of both groups presented in the corresponding forest plot.

### Efficacy outcomes

#### Primary efficacy criterion: National Institutes of Health Stroke Scale

**Fig. 1:**
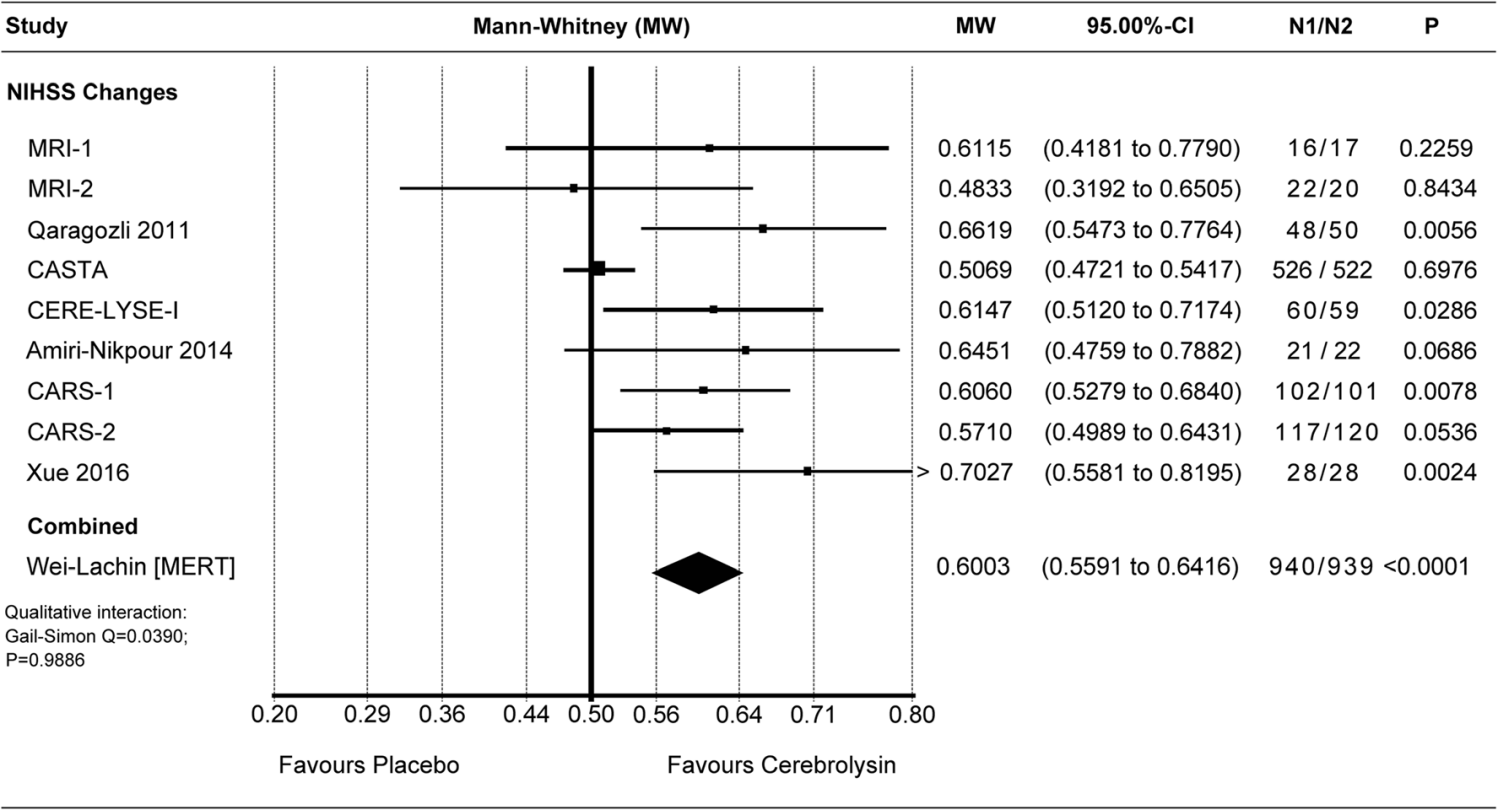
Meta-analysis of NIHSS changes from baseline. Comparison of Cerebrolysin (30 ml/day) versus placebo at day 30 (or 21) in the ITT population; LOCF. Wei-Lachin pooling procedure (MERT), effect size: Mann-Whitney (MW)

The primary NIHSS analysis at day 30 (or 21), based on nine RCTs, showed a more than “small” superiority of Cerebrolysin as compared to placebo (MW 0.60; 95% CI 0.56 to 0.64; Fig. 1). The combined result of the Mann-Whitney effect size measure was statistically significant (*P* < 0.0001, *N* = 1879; Wei-Lachin pooling procedure [MERT]). The individual effect sizes showed more than “small” to “medium-sized” superiority of Cerebrolysin in seven out of nine RCTs, with stand-alone statistical significance in four individual trials.

Clinical relevance was evaluated applying the original NINDS definition [30] (NIHSS change of at least 4 points or resolution of symptoms). The result of the corresponding meta-analysis, based on the OR at day 30 (or 21), was statistically significant in favor of Cerebrolysin (*P*_FixedEffect_ 0.03, *P*_RandomEffects_ 0.04, Online Supplement, Fig. X2). The combined rate difference regarding clinically relevant NIHSS changes was 12.9%, favoring Cerebrolysin (95% CI 6.7% to 19.2%, see Online Supplement, Fig. X3). The combined number needed to treat (NNT) for clinically relevant changes in early NIHSS was 7.7 (95% CI 5.2 to 15.0).

#### Long-term results in moderate-severe population

**Fig. 2:**
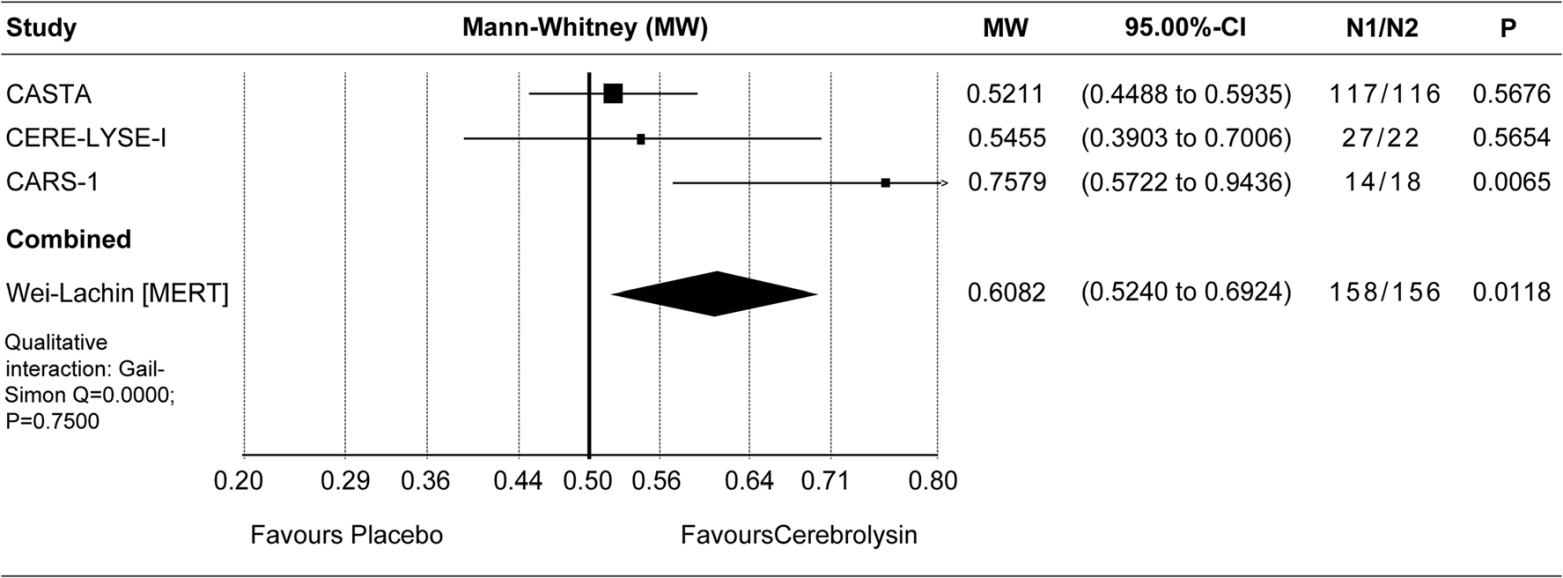
Meta-analysis of mRS at day 90 in patients with baseline NIHSS > 12. Comparison of Cerebrolysin (30 ml/day) versus placebo in the ITT population; LOCF. Wei-Lachin pooling procedure (MERT), effect size: Mann-Whitney (MW)

**Fig. 3:**
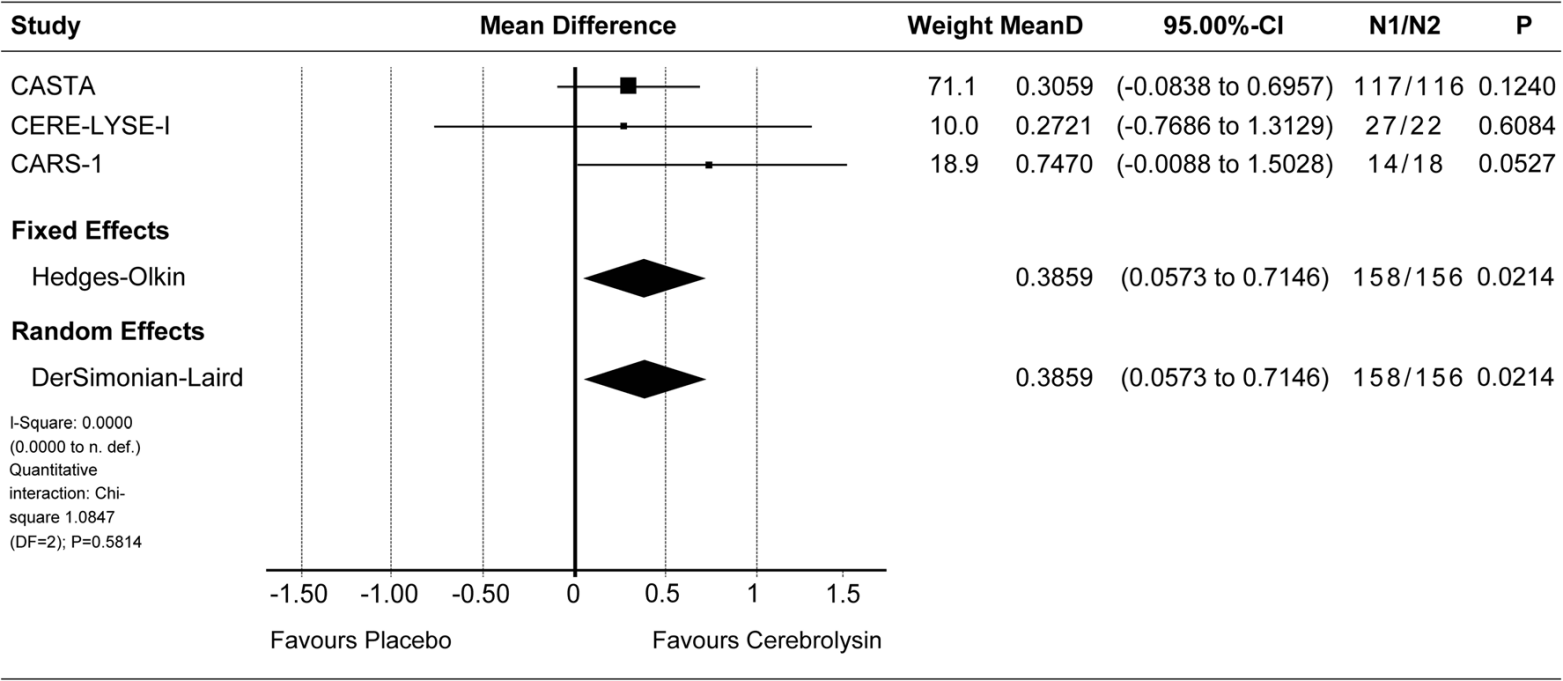
Meta-analysis of mRS at day 90 in patients with baseline NIHSS > 12. Comparison of Cerebrolysin (30 ml/day) versus placebo in the ITT population; LOCF. Analysis of Covariance. “Classic” fixed effect and random effects analysis, effect size: mean difference

Final global disability was evaluated by means of the mRS at day 90. Due to the baseline heterogeneity of the included trials and due to expected floor effects in studies with very mild stroke, the long-term analysis was performed for the clinically relevant moderate-severe population of patients with baseline NIHSS greater than 12 (pre-defined subgroup of the CASTA trial) [5]. The pre-planned nonparametric full-scale ordinal analysis of mRS at day 90 was performed in three trials with *N* ≥ 10 in this subgroup and resulted in statistical significance in favor of Cerebrolysin (MW 0.61, 95% CI 0.52 to 0.69, *P* = 0.01, *N* = 314; Wei-Lachin pooling procedure (MERT); Fig. 2). The parametric sensitivity analysis, based on analyses of covariance (ANCOVA) with baseline NIHSS as covariate, resulted in an adjusted mRS mean difference of 0.39 in favor of Cerebrolysin in both fixed effect and random effects model, with *P* = 0.02 and 95% CI 0.06 to 0.71 (*I* ^2^ = 0%; Fig. 3). The results of the parametric and nonparametric approach are well comparable, indicating robustness of the mRS evaluation. It should be noted that all included trials were evaluated with IPD, which is regarded as the gold standard for synthesizing evidence across clinical studies [13].

### Safety and tolerability

**Fig. 4:**
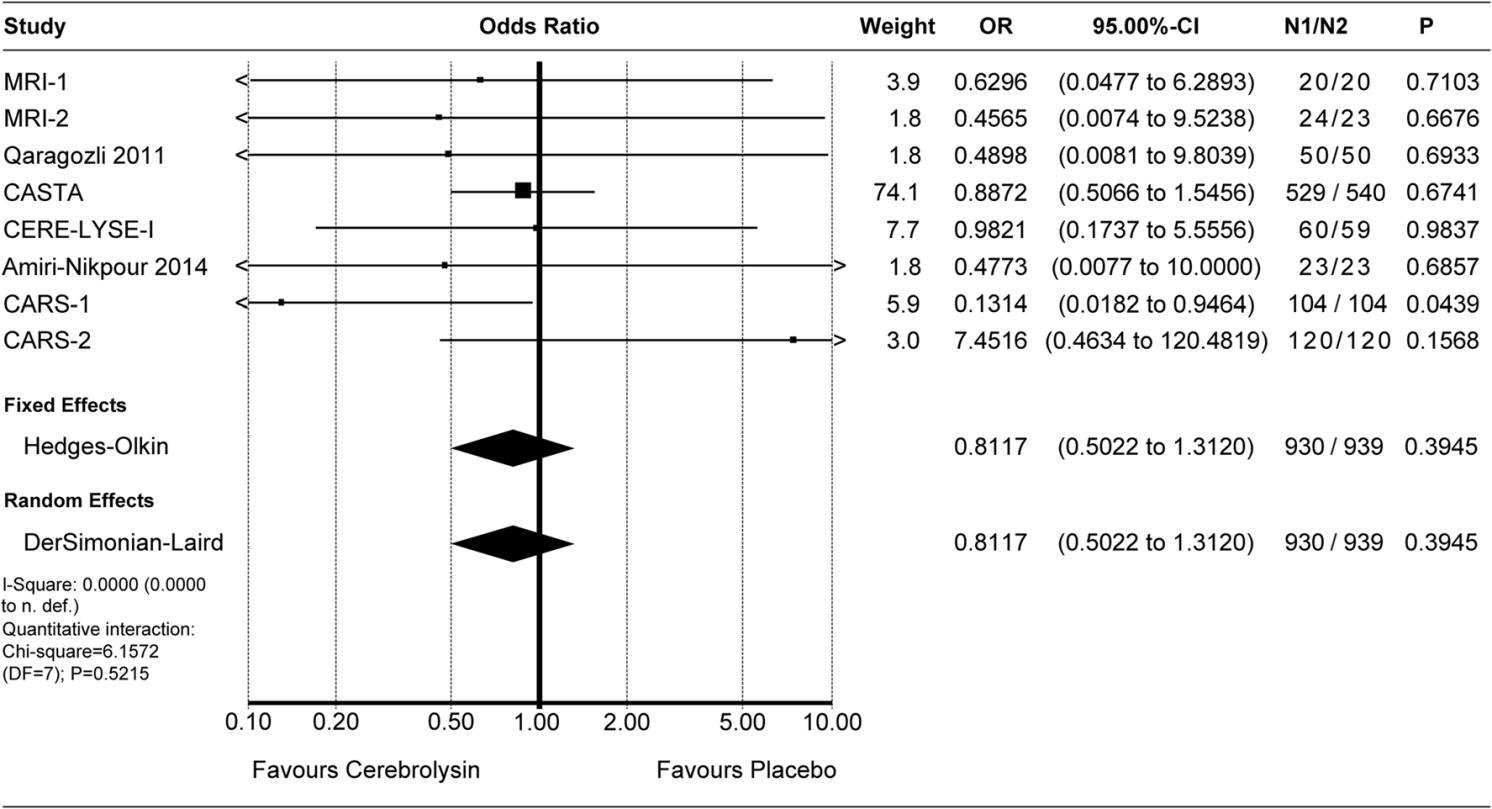
Deaths (all cause). Comparison of Cerebrolysin (30 ml/day) versus placebo in the safety population. “Classic” fixed effect and random effects analysis, effect size: odds ratio (OR)

Deaths were evaluated by means of the OR. The combined OR for deaths of all cause was resulting in a small superiority of Cerebrolysin, which was statistically not significant (OR 0.81, *P* 0.39, Fig. 4). Due to the low incidence rates of deaths (< 10%), the additionally calculated RR was close to the OR (combined RR 0.83, *P* 0.38, see Online Supplement, Fig. X7).

Crude pooling of deaths across studies resulted in a total of 39 deaths out of 958 subjects treated with Cerebrolysin (4.1%), as compared to 49 deaths out of 968 subjects treated with placebo (5.1%).

**Fig. 5:**
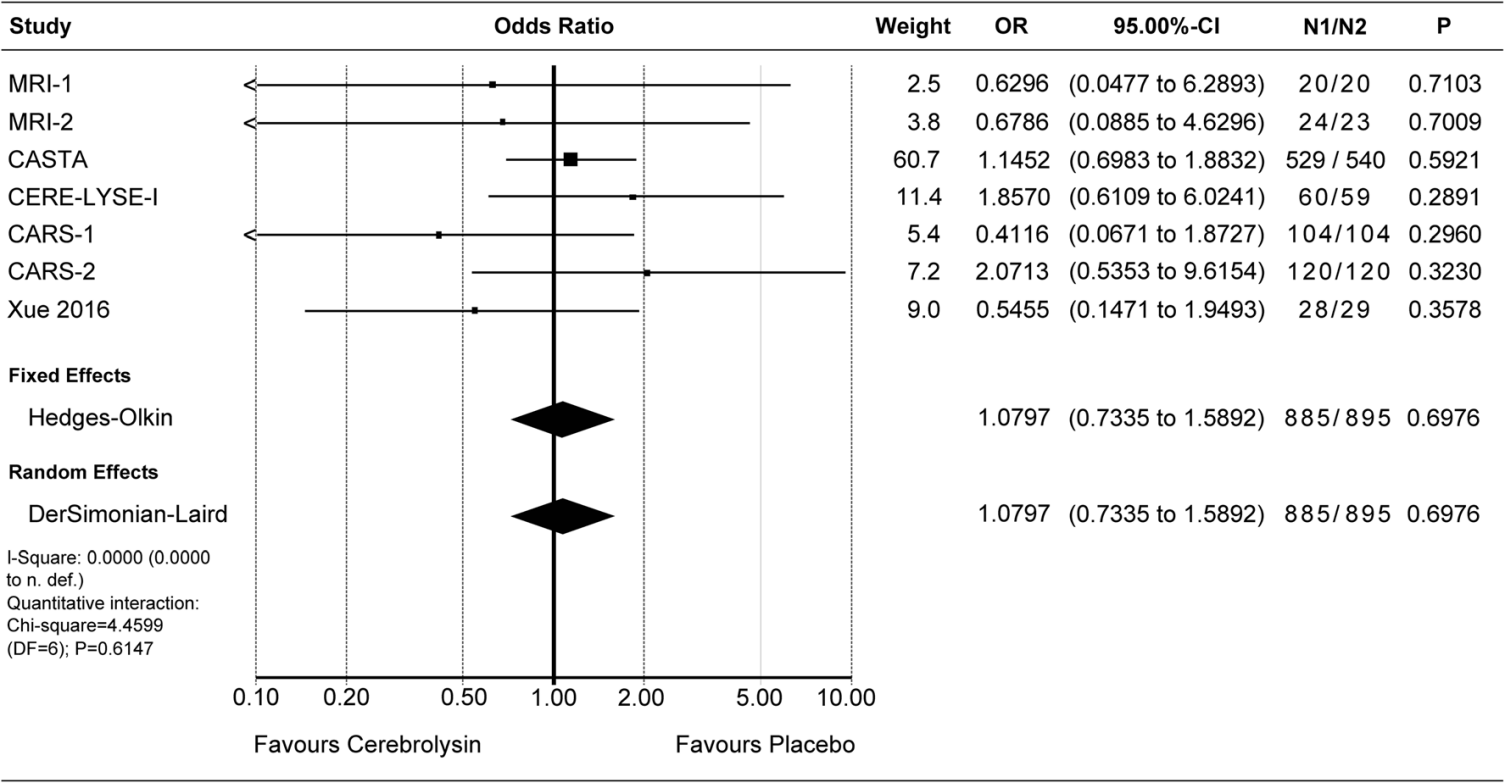
Patients with at least one serious adverse event (TESAE). Comparison of Cerebrolysin (30 ml/day) versus placebo in the safety population. “Classic” fixed effect and random effects analysis, effect size: odds ratio (OR)

Serious adverse events were reported in 75 out of 885 subjects treated with Cerebrolysin (8.5%), and in 72 out of 895 subjects treated with placebo (8.1%). In two studies, no information on SAE was available [4, 11]. The combined OR of the formal meta-analysis indicates marginal differences only (OR 1.08, *P* 0.70, Fig. 5). Also, the RR for serious adverse events is close to equality (RR 1.04, *P*_FixedEffect_ 0.80, *P*_RandomEffects_ 0.82, Online Supplement, Fig. X8).

**Fig. 6:**
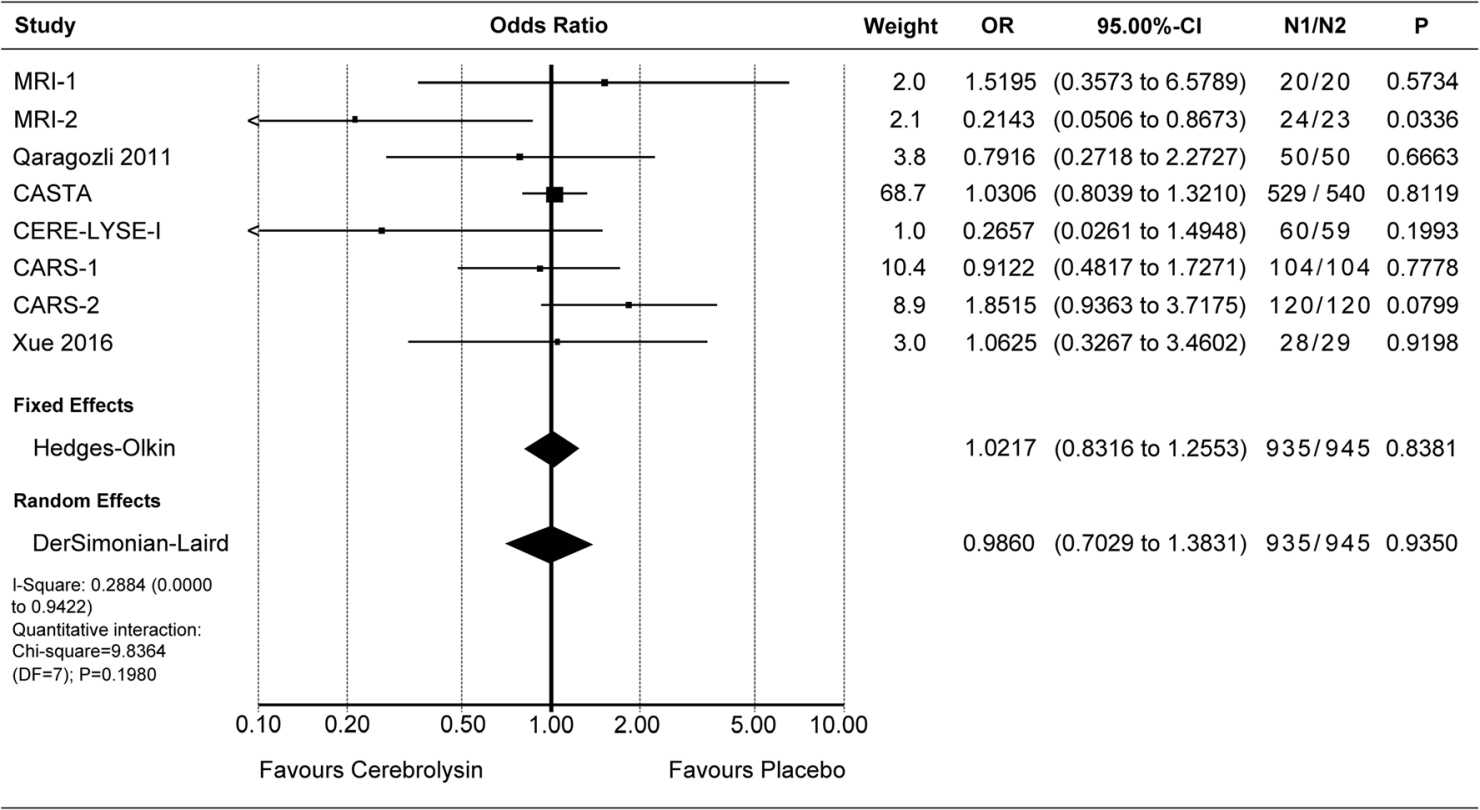
Patients with at least one adverse event (TEAE). Comparison of Cerebrolysin (30 ml/day) versus placebo in the safety population. “Classic” fixed effect and random effects analysis, effect size: odds ratio (OR)

At least one adverse event was reported in 435 out of 935 subjects in the Cerebrolysin group (46.5%), and in 438 out of 945 subjects in the placebo group (46.3%). In one study, no information on adverse events was available [11]. The formal meta-analysis resulted in a combined OR_FixedEffect_ of 1.02 and OR_RandomEffects_ of 0.99, thus, varying around the benchmark of equality (Fig. 6). The corresponding risk ratios were 0.96 for fixed effect and 0.98 for random effects (*P*_FixedEffect_ 0.28, *P*_RandomEffects_ 0.68, Online Supplement, Fig. X9).

All in all, the safety outcome reflected the expected safety and tolerability of patients after acute ischemic stroke. With a slight superiority regarding deaths (OR 0.81; Fig. 4), and marginal differences regarding TEAE and TESAE (OR 1.02/0.99, Fig. 6; OR 1.08, Fig. 5), Cerebrolysin can be considered well tolerable and comparable to placebo. The *P* values for all OR comparisons were > 0.3, regardless of the chosen model.

### Sensitivity analysis

In addition to the pre-defined Lachin pooling procedure (MERT), which is comparably robust with respect to heterogeneity of included trials, “classic” fixed and random effects models were calculated as sensitivity analyses. The results are well comparable to the primary analysis, showing again a statistically significant superiority of Cerebrolysin as compared to placebo (*Fixed Effects*: MW 0.55; 95% CI 0.53 to 0.58; *P* = 0.0001; *Random Effects:* MW 0.59; 95% CI 0.54–0.64; *P* = 0.0005; *I* ^2^ = 0.62; Online Supplement, Fig. X4).

*Leave*-*one*-*out analysis* is an important tool to verify the robustness of the results. Applying this method, each study is successively excluded from the main analysis, resulting in as many meta-analyses as there are participating studies. All nine *leave*-*one*-*out analyses* on NIHSS turned out to be statistically significant. This does not only apply to the first-line analysis (Wei-Lachin pooling procedure [MERT]; Online Supplement, Fig. X5, left panel) but also apply to the corresponding sensitivity analyses by means of “classic” fixed effects and random effects models (all *P*_leave-one-out_ < 0.05; Online Supplement, Fig. X5, right panel). This is a strong sign for an overall robustness of the results.

## Discussion

Our meta-analysis comprised a total of 1879 patients and showed the superiority of Cerebrolysin compared to placebo on both neurological deficits, measured by NIHSS, and functional outcome, measured by mRS.

The importance of the baseline severity for assay sensitivity of stroke trials and for prediction of patient outcome after stroke was highlighted by many researchers [31–33]. DeGraba et al. [33] found that 45 % (40/88) of those with an initial NIHSS score of < 8 were functionally normal at 48 h, whereas only 2.4% (1/41) of those with NIHSS scores of ≥ 8 returned to a normal examination within this period. These mild cases, quickly gaining functional normality, might introduce substantial floor effects, preventing detection of group differences in clinical trials. On individual study level, this issue was already addressed in several Cerebrolysin publications [5, 8].

**Fig. 7:**
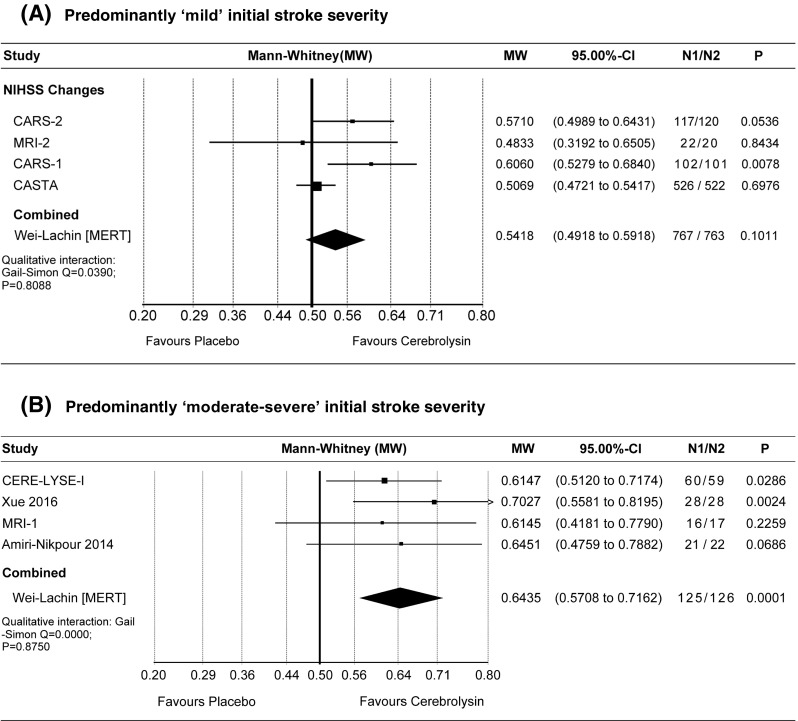
Meta-analysis of early NIHSS changes in predominantly mild (**a**) and moderate-severe patients (**b**). Comparison of Cerebrolysin (30 ml/day) at day 30 (or 21); ITT; LOCF. Wei-Lachin pooling procedure (MERT), effect size: Mann-Whitney (MW)

In order to assess the impact of initial stroke severity on observable effect sizes based on a broader ensemble of studies, a meta-analysis was conducted comparing the subset of studies with the highest baseline NIHSS scores to the studies with the lowest baseline NIHSS scores (each subset included four studies; the trial just in the middle, Qaragozli 2011 [4] with an overall NIHSS mean of 10, was left out in order to avoid arbitrary cutoff). Figure 7 shows the results of the two subsets of stroke severity.

As shown in Fig. 7, the combined effect size for the milder ensemble was 0.54 (A) while the effect size for the more severe ensemble was 0.64 (B), thus, revealing substantially larger treatment effects in the more severe subset (see also the results of the “classic” meta-analyses with MW_FixedEffect_ 0.53/MW_RandomEffects_ 0.55 and *I* ^2^ 54% in the “mild” subset, as compared to MW_FixedEffect_ 0.64/MW_RandomEffects_ 0.64 with *I* ^2^ 0% in the homogeneous “severe” subset, see Online Supplement, Fig. X6).

The smaller effects in the subset with low initial stroke severity are well explained by floor effects due to milder stroke. It is interesting to note that all four studies with the more severe patients showed larger effect sizes than any study with predominantly milder cases. This consistent finding is supporting the findings of DeGraba et al. [33] and others on the importance of the NIHSS baseline severity for adequately designing future stroke trials.

There is a positive benefit-risk relation in favor of Cerebrolysin with a statistically significant superiority as compared to placebo regarding the stroke-specific outcome, while the safety profile was comparable to placebo with a tendency for reduction of deaths (RR 0.83).

A previous meta-analysis of Cerebrolysin in acute ischemic stroke [34] was based only on mortality as primary efficacy endpoint. However, with an overall death rate below 10% in both groups, this approach was lacking assay sensitivity and other clinical outcomes were not involved in the analyses. The authors’ conclusion that the findings “do not demonstrate clinical benefits of cerebrolysin for treating acute ischaemic stroke” may be regarded as overstated since the outcome of the more than 90% survivers was neglected in this meta-analysis (there were mostly mild to moderate stroke patients with mean baseline NIHSS below 12). In addition, regarding deaths, invalid values were taken from one of the study publications: study Skvortsova 2004 [9] was mistakenly evaluated by the reviewers with seven deaths in the combined Cerebrolysin groups of 10 and 50 ml instead of the reported five deaths. Also for serious adverse events, incorrect values were taken from publications [35], which was meanwhile corrected by the authors [34]. A post hoc analysis of the same authors on non-fatal serious adverse events, based on three studies and resulting in “moderate-quality evidence of an increase in non-fatal SAEs with cerebrolysin,” could not be confirmed with the present larger study ensemble (all *P* > 0.1 with identical procedures as applied by the review authors, see Online Supplement, Fig. X10).

Another recent meta-analysis resulting in “no significant efficacy on the neurological functional recovery” was based on dichotomization of mRS, NIHSS, and BI at day 90, i.e., not on full outcome scales [36]. As highlighted by leading researchers and methodologists, dichotomization of a full scale is burdened with loss of power and arbitrary choice of cutoffs [22, 37, 38], allowing only limited statements on treatment effects. The cited meta-analyses included only three studies and the validity of the dichotomized NIHSS results was limited by severe heterogeneity (*I* ^2^ = 87%) and poor precision of the chosen random effects model [39–41] (it should be noted that the same limitation applies also to another recent meta-analysis [42], combining only two, at a maximum three trials by means of random effects models).

The authors conceded that “all of the included trials showed accelerated recovery at early time points of assessment” and suggested that the “unexpected mildly affected patients in these studies might be the main cause of the inconclusive results”. In fact, this statement is confirmed by the present larger full-scale meta-analysis on NIHSS, providing conclusiveness for accelerated recovery at early time points (Fig. 1) while demonstrating the masking of treatment effects in patients with mild stroke severity (Fig. 7a vs. b).

### Strengths

The compelling strength of the current meta-analysis is the homogeneity and consistent superiority of Cerebrolysin regarding early neurological function. With four stand-alone statistically significant trials and a combined overall *P* < 0.0001, based on 1879 included patients, there is strong evidence for early beneficial clinical effects of Cerebrolysin. A further strength is the largest number of included Cerebrolysin studies so far, and the use of individual patient data from the majority of the trials (IPD analysis). All sensitivity analyses supported the main result and leave-one-out analyses demonstrated the robustness of the positive findings across analysis pathways.

### Limitations

There was a large heterogeneity of the trials with respect to baseline stroke severity: NIHSS trial medians were reaching from 7 to 14, thus including trials with very little assay sensitivity for long-term outcome. While assay sensitivity was sufficient for early benefit, studies with rather mild cases failed to demonstrate functional benefit at day 90. Another limitation is the restricted information on study conduct from some of the included trials. Analysis for publication bias showed no tendency for overreporting *positive* trials; however, the study CASTA was identified as *negative* outlier in the funnel plot. Patient-centered outcomes were not available in most stroke trials, and inclusion of more subtle outcome measures as cognitive functioning, health-related quality of life, or social participation is recommended for future trials. The same applies to the inclusion of prolonged observation (6 months, 1 year).

## Summary

A recent meta-analysis of two trials on Cerebrolysin after acute ischemic stroke showed significant positive results on early neurological improvements [2]. The current meta-analysis showed:

- Nonparametric MW effect size on the National Institutes of Health Stroke Scale on day 30 (or 21), combining the results of nine RCTs, indicated superiority of Cerebrolysin as compared with placebo (MW 0.60, *P* < 0.0001, *N* = 1879).
- The strongest effects were observed in trials with high baseline stroke severity as expressed by NIHSS (MW = 0.64; *P* = 0.0001).
- The combined NNT for clinically relevant changes in early NIHSS was 7.7 (95% CI 5.2 to 15.0).
- The pre-planned full-scale ordinal analysis of mRS at day 90 in the moderate to severe patients resulted in MW 0.61 with statistical significance in favor of Cerebrolysin (95% CI 0.52 to 0.69, *P* = 0.0118, *N* = 314; Wei-Lachin pooling procedure [MERT]).
- All sensitivity analyses supported the first-line results.

With respect to safety aspects, Cerebrolysin was well comparable to placebo, with a tendency to reduction of deaths (RR 0.83). With respect to TESAE and TEAE, only marginal group differences were found (all *P* > 0.1). The crude overall rate of patients with events was 39 vs. 49 for deaths, 75 vs. 72 for SAE, and 435 vs. 438 for AE. All in all, Cerebrolysin is showing a favorable benefit/risk ratio, providing a promising option for early treatment after acute ischemic stroke. Further clinical trials are required to provide sufficient evidence also after discharge (day 90) and with longer, repetitive treatment cycles.

## Conclusions

Our meta-analysis confirms previous evidence that Cerebrolysin has a beneficial effect on early global neurological deficits in patients with acute ischemic stroke. Moreover, our results showed a clinically relevant and significant improvement of functional outcome at day 90 based on the mRS in the moderate to severe group.

## Electronic supplementary material

ESM 1 (DOCX 1956 kb)
