# Supplementary material for: Safety and efficacy of Cerebrolysin in early post-stroke recovery: a meta-analysis of nine randomized clinical trials
Source: Neurol Sci. 2017 Dec 16;39(4):629–40. doi: 10.1007/s10072-017-3214-0 (PMC5884916; doi:10.1007/s10072-017-3214-0)
Supplement: Supplementary file 1 — (DOCX 1956 kb) [file 10072_2017_3214_MOESM1_ESM.docx]

**ONLINE SUPPLEMENT**


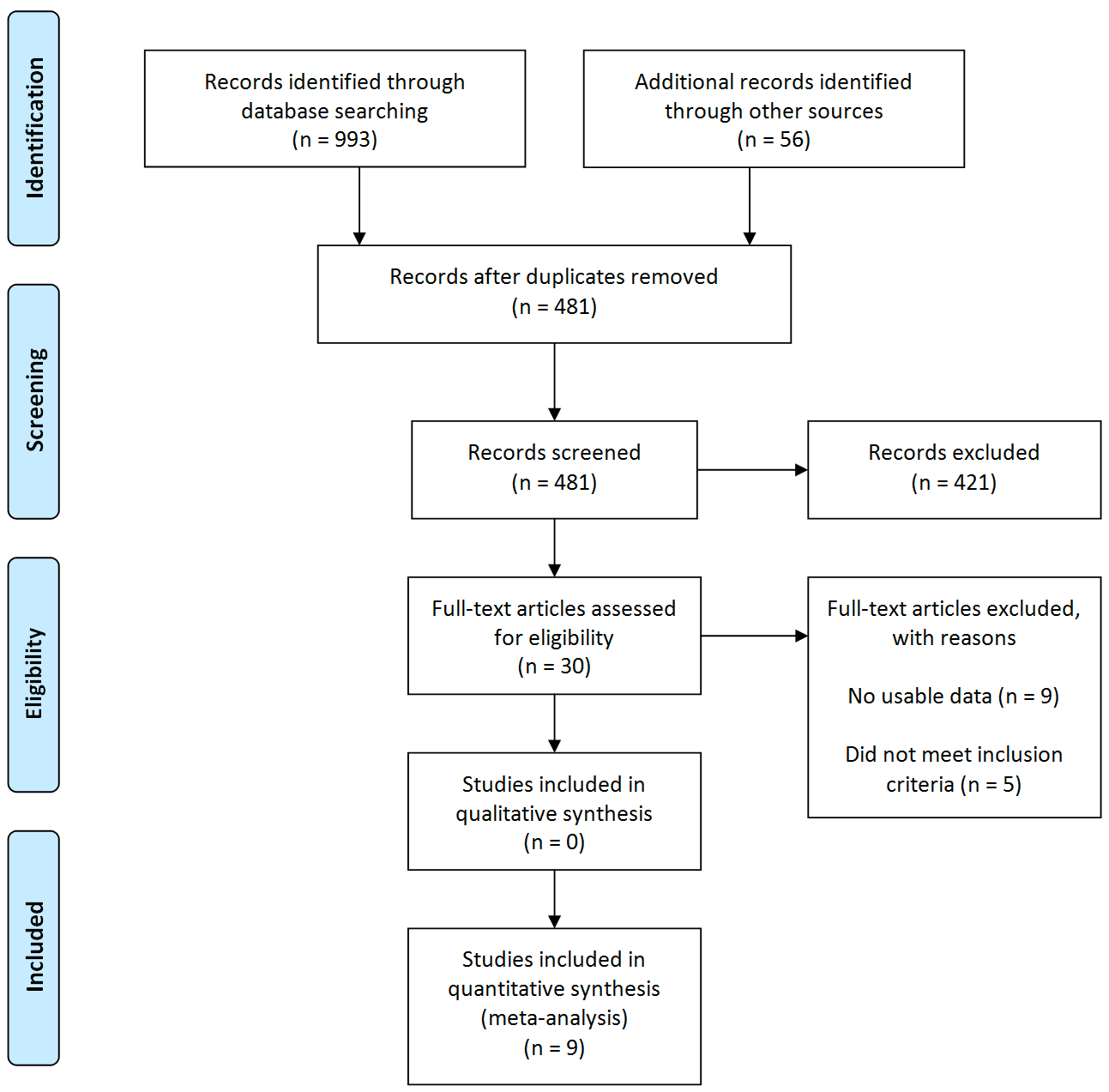


**Figure X1.** Flow chart of study selection.

| **Methods of Data Extraction and Supplemental Pathways** |
| --- |
| 1. Aggregate data were extracted from eligible full-text articles and, where applicable, from clinical study reports by one independent person and entered on high security PCs (HSPC) within the validated working environment at the department ‘Clinical Research/Biometry’ in the institute IDV Data Analysis and Study Planning (Krailling/Germany). 2. Data entries were validated by another two independent persons at IDV, including a full internal source documentation and source verification. 3. One multicenter trial (MRI-1) was preliminarily published in Russia for Russian sites only [1], however, for the present meta-analysis all sites were included capturing the integrated data from the final clinical study report (CSR). 4. For study Xue 2016 [2], raw data of dropouts were provided by the authors in order to allow LOCF analysis. 5. For the safety evaluations of study CASTA, the finalized CASTA safety database was used. |

**Table X2. Data Extraction**

| Early Neurological Improvement: The National Institutes of Health Stroke Scale (NIHSS) |
| --- |
| The original outcome scale available in all nine selected studies was the National Institutes of Health Stroke Scale (NIHSS) at day 30 (or 21) [3]. The NIHSS reflects neurological impairment, the clinical domain in which early effects of acute stroke therapies are likely to be most marked^42^. Recent research showed that the NIHSS in fact is most sensitive for such early points in time. Furthermore, NIHSS is less influenced by extraneous factors, improving sensitivity to acute treatment effects [4]. The NIHSS is a non-linear ordinal scale, ranging from 0 (no neurological symptoms) to 42 (very severe neurological deficits). The NIHSS scores were evaluated as changes from baseline at day 30 (or 21) by means of the robust Mann-Whitney (MW) effect size measure, which is the recommended ES for full scale ordinal analysis [5]. The MW effect sizes were calculated from individual patient data (IPD) in 5 out of 9 trials. For aggregate data, MW was interconverted from standardized mean difference via relation MW = Φ (d/√2), with Φ (∙) meaning the standard normal distribution and d being the standardized difference [6, 7]. For one study [8] the MW was calculated from absolute NIHSS values since no changes from baseline were available; the corresponding baseline levels were identical in both treatment groups, suggesting good validity of the substitute. |
| Final Global Disability: Modified Rankin Scale |
| The analysis of this secondary endpoint was performed for evaluation of the long-term outcome (day 90). The modified Rankin Scale [9] (mRS) is a functional global outcome scale measuring the level of disability after a stroke. It is a 7-point ordinal scale with a score of 0 indicative of no residual symptoms and the worst possible score of 6, which is assigned in case of death. The analysis was based on final changes from baseline (pre-planned nonparametric analysis) as well as on final scores with adjustment for NIHSS baseline severity. |

**Table X3. Outcome Scales**

| Statistical Methods of Synthesis |
| --- |
| The method of synthesis for the Mann-Whitney (MW) effect size measure [5, 7 10, 11, , 12] and for the RD was the Wei-Lachin test of stochastic ordering (one-dimensional test) [13], a maximin-efficient robust test (MERT) [14, 15] which provides a combined MW estimate and test of overall treatment effect from an ensemble of independent studies. In combination with stochastic ordering or one-dimensional alternative it is a powerful and robust meta-analytic procedure for combining the MW effect size across studies.  This method is identical with the pre-planned procedures of the previously published meta-analysis on the two CARS trials [16]. The one-dimensional alternative of stochastic superiority is to be interpreted as follows: at least one trial has an underlying true beneficial effect and none have an adverse effect (no qualitative interaction). This approach is ‘assumption-free’ and has been shown to be robust also with respect to presence of heterogeneity [13]. Qualitative interaction was tested by means of the Gail-Simon test [17], with P-values < 0.10 preventing formal combination of studies.  As sensitivity analysis the “classic” approaches based on fixed effects model (Hedges-Olkin) [18] and random effects model (DerSimonian-Laird) [19] were calculated. The fixed effects model assumes one single (‘fix’) underlying “true” effect. Opposed to this, the common random effects model (DerSimonian-Laird) assumes a normal distribution of a bundle of “true” effects. While some researchers don’t accept the fixed effects model in case of heterogeneity or focus a priori on the random effects model, it is difficult to establish the validity of the normality assumption, a major disadvantage of the random effects model. All in all, corresponding methodology is still in discussion and there is no consensus on how to best approach “classic” synthesis for ordinal data such as the nonlinear NIHSS. Associated tests for quantitative heterogeneity were performed using standard chi-square statistic [20] and I^2^ statistic [21].  The analyses were performed using the software packages TESTIMATE and METASUB on high security PCs (HSPC) within a validated working environment at the department ‘Clinical Research/Biometry’ in the institute IDV Data Analysis and Study Planning (Krailling/Germany) under supervision of Volker W. Rahlfs, PhD., C. Stat. (RSS), with a ’Certificate Biometry in Medicine GMDS’. Safety sensitivity analyses based on risk ratios (RR) were performed using The Cochrane Collaboration Review Manager (Revman Version 5.3), in order to allow comparison of the results of the present larger study ensemble with previous external results including substantially smaller ensembles. |

**Table X4. Methods of Synthesis**

**Risk of bias within studies**

| Bias  Study | Selection | | Performance | Detection | Attrition | Reporting | Other |
| --- | --- | --- | --- | --- | --- | --- | --- |
|  | Appropriate generation of the allocation sequence | Concealment of the allocation sequence | Blinding of participant and health care providers | Blinding of outcome assessors | Assessment of incomplete outcome data | Selective outcome reporting | Other biases |
| MRI-1 | Low risk | Low risk | Low risk | Low risk | Low risk | Low risk | Low risk |
| MRI-2 | Low risk | Low risk | Low risk | Low risk | Low risk | Low risk | Low risk |
| Qaragozli 2011 | Low risk | Unclear risk | Unclear risk | Unclear risk | Low risk | Low risk | Low risk |
| Cere-Lyse-I | Low risk | Low risk | Low risk | Low risk | Low risk | Low risk | Low risk |
| CASTA | Low risk | Low risk | Low risk | Low risk | Low risk | Low risk | Low risk |
| Amiri-Nikpour et al. 2014 | Unclear risk | Unclear risk | Unclear risk | Unclear risk | Low risk | Low risk | Low risk |
| CARS-1 | Low risk | Low risk | Low risk | Low risk | Low risk | Low risk | Low risk |
| CARS-2 | Low risk | Low risk | Low risk | Low risk | Low risk | Low risk | Low risk |
| Xue et al. 2016 | Low risk | Low risk | Unclear risk | Unclear risk | Low risk | Low risk | Low risk |
| Risk of bias assessment was performed using all available data (publications, clinical study reports, individual patient data files, feedbacks from authors). Attrition assessment was based on the primary point in time of this meta-analysis (21 or 30 days), not on later follow-up visits. For some studies there was insufficient information available to permit judgment of all risks of bias. | | | | | | | |

**Table X5. Risk of bias**

**Figure X2.** Meta-analysis of NIHSS clinically relevant changes from baseline (≥ 4). Comparison of Cerebrolysin (30 ml/day) versus placebo at day 30 (or 21) in the ITT population; LOCF. ‘Classic’ fixed effect and random effects analysis, effect size: Odds Ratio (OR).

**Figure X3.** Meta-analysis of NIHSS clinically relevant changes from baseline (≥ 4). Comparison of Cerebrolysin (30 ml/day) versus placebo at day 30 (or 21) in the ITT population; LOCF. Wei-Lachin pooling procedure (MERT), effect size: Rate Difference (RD).

**Figure X4.** Meta-analysis of NIHSS changes from baseline. Comparison of Cerebrolysin (30 ml/day) versus placebo at day 30 (or 21) in the ITT population; LOCF. ‘Classic’ fixed effect and random effects analysis, effect size: Mann-Whitney (MW).

**Figure X5.** Leave-One-Out meta-analysis of NIHSS changes from baseline. Comparison of Cerebrolysin (30 ml/day) versus placebo at day 30 (or 21) in the ITT population; LOCF. Left panel: Wei-Lachin pooling procedure (MERT), effect size: Mann-Whitney (MW). Right panel: ‘Classic’ fixed effect and random effects analysis, effect size: Mann-Whitney (MW).

**(A) Predominantly ‘mild’ initial stroke severity**

**(B) Predominantly ‘moderate-severe’ initial stroke severity**

Figure X6. Meta-analysis of early NIHSS changes in predominantly mild (A) and moderate-severe patients (B). Comparison of Cerebrolysin (30 ml/day) at day 30 (or 21); ITT; LOCF. ‘Classic’ fixed effect and random effects analysis, effect size: Mann-Whitney (MW).

**Figure X7.** Deaths (all cause). Comparison of Cerebrolysin (30 ml/day) versus placebo in the safety population. ‘Classic’ fixed effect (upper panel) and random effects (lower panel) analysis, effect size: Odds Ratio (OR).

**Figure X8.** Patients with at least one serious adverse event (TESAE). Comparison of Cerebrolysin (30 ml/day) versus placebo in the safety population. ‘Classic’ fixed effect and random effects analysis, effect size: Odds Ratio (OR).

**Figure X9.** Patients with at least one adverse event (TEAE). Comparison of Cerebrolysin (30 ml/day) versus placebo in the safety population. ‘Classic’ fixed effect and random effects analysis, effect size: Odds Ratio (OR).

**Figure X10.** Patients with at least one non-fatal serious adverse event (non-fatal TEAE). Comparison of Cerebrolysin (30 ml/day) versus placebo in the safety population. ‘Classic’ fixed effect and random effects analysis, effect size: Odds Ratio (OR).

**References**

[1] Skvortsova, V. I., Stakhovskaia, L. V., Gubskiĭ, L. V., Shamalov, N. A., Tikhonova, I. V., & Smychkov, A. S. (2003). A randomized, double-blind, placebo-controlled study of Cerebrolysin safety and efficacy in the treatment of acute ischemic stroke. Zhurnal nevrologii i psikhiatrii imeni SS Korsakova/Ministerstvo zdravookhraneniia i meditsinskoi promyshlennosti Rossiiskoi Federatsii, Vserossiiskoe obshchestvo nevrologov [i] Vserossiiskoe obshchestvo psikhiatrov, (Suppl 11), 51-55.

[2] Xue, L. X., Zhang, T., Zhao, Y. W., Geng, Z., Chen, J. J., & Chen, H. (2016). Efficacy and safety comparison of DL-3-n-butylphthalide and Cerebrolysin: Effects on neurological and behavioral outcomes in acute ischemic stroke. Experimental and therapeutic medicine, 11(5), 2015-2020.

[3] Brott TG, Adams HP, Olinger CP, Marler JR, Barsan WG, Biller J, Spilker J, Holleran R, Eberle R, Hertzberg V, et al (1989). Measurements of acute cerebral infarction: a clinical examination scale. Stroke; 20: 864–870.

[4] Kerr DM, Fulton RL, Lees KR. (2012). Seven-Day NIHSS Is a Sensitive Outcome Measure for Exploratory Clinical Trials in Acute Stroke. Stroke;43:1401-1403.

[5] Rothmann, M. D., Wiens, B. L., & Chan, I. S. (2011). Design and analysis of non-inferiority trials. CRC Press.

[6] Colditz, G. A., Miller, J. N., & Mosteller, F. (1988). Measuring gain in the evaluation of medical technology The probability of a better outcome. International journal of technology assessment in health care, 4(4), 637-642.

[7] Rahlfs, V. W., Zimmermann, H., & Lees, K. R. (2014). Effect size measures and their relationships in stroke studies. Stroke, 45(2), 627-633.

[8] Amiri-Nikpour, M. R., Nazarbaghi, S., Ahmadi-Salmasi, B., Mokari, T., Tahamtan, U., & Rezaei, Y. (2014). Cerebrolysin effects on neurological outcomes and cerebral blood flow in acute ischemic stroke. Neuropsychiatric disease and treatment, 10, 2299.

[9] van Swieten J, Koudstaal P, Visser M, Schouten H, et al. (1988). Interobserver agreement for the assessment of handicap in stroke patients. Stroke.;19(5):604–607.

[10] D'Agostino, R. B., Campbell, M., & Greenhouse, J. (2006). The Mann–Whitney statistic: continuous use and discovery. Statistics in Medicine, 25(4), 541-542.. doi: 10.1002/sim.2508.

[11] Munzel, U., & Hauschke, D. (2003). A nonparametric test for proving noninferiority in clinical trials with ordered categorical data. Pharmaceutical Statistics, 2(1), 31-37. doi: 10.1002/pst.17.

[12] Kieser M, Friede T, Gondan M. (2013). Assessment of statistical significance and clinical relevance. Stat Med.;32:1707–1719.

[13] Lachin, J. M. (2009). Biostatistical methods: the assessment of relative risks (Vol. 509). John Wiley & Sons.

[14] Frick, H. (1994). A maxmin linear test of normal means and its application to lachin’s data. Communications in statistics-theory and methods, 23(4), 1021-1029.

[15] Frick, H. (1995). Comparing Trials with Multiple Outcomes: The Multivariate One‐Sided Hypothesis with Unknown Covariances. Biometrical journal, 37(8), 909-917.

[16] Guekht, A. et al. (2017). Safety and efficacy of Cerebrolysin in motor function recovery after stroke: a meta-analysis of the CARS trials. Neurological Sciences, 1-9.. DOI: 10.1007/s10072-017-3037-z.

[17] Gail M, Simon R. Testing for qualitative interactions between treatment effects and patient subsets. Biometrics 1985; 41: 361-372.

[18] Hedges, L.V., Olkin, J. (1985). Statistical Methods for Meta-Analysis, Academic Press Inc., San Diego.

[19] DerSimonian, R., & Laird, N. (1986). Meta-analysis in clinical trials. Controlled clinical trials, 7(3), 177-188.

[20] Higgins, J. P., Thompson, S. G., Deeks, J. J., & Altman, D. G. (2003). Measuring inconsistency in meta-analyses. BMJ: British Medical Journal, 327(7414), 557.

[21] Higgins, J., & Thompson, S. G. (2002). Quantifying heterogeneity in a meta‐analysis. Statistics in medicine, 21(11), 1539-1558.
